# Beyond Cell Death – Antiapoptotic Bcl-2 Proteins Regulate Migration and Invasion of Colorectal Cancer Cells *In Vitro*

**DOI:** 10.1371/journal.pone.0076446

**Published:** 2013-10-03

**Authors:** Bruno Christian Koehler, Anna-Lena Scherr, Stephan Lorenz, Toni Urbanik, Nicole Kautz, Christin Elssner, Stefan Welte, Justo Lorenzo Bermejo, Dirk Jäger, Henning Schulze-Bergkamen

**Affiliations:** 1 National Center for Tumor Diseases, Department of Medical Oncology, Internal Medicine VI, Heidelberg University Hospital, Heidelberg, Germany; 2 Institute of Medical Biometry and Informatics, University Hospital Heidelberg, Heidelberg, Germany; Sun Yat-sen University Medical School, China

## Abstract

Migration and invasion of malignant cells are prerequisites for cancer progression and metastasis. The Bcl-2 family of proteins consists of about 25 members and has been extensively studied in the context of apoptosis. Despite the fact that small molecules targeting Bcl-2 proteins have already entered clinical trials, very few studies investigated a role of antiapoptotic Bcl-2 proteins beside cell death in the context of metastasis. The aim of this study was to dissect a potential role of the antiapoptotic Bcl-2 proteins Mcl-1, Bcl-2 and Bcl-x_L_ on migration and invasion of colorectal cancer cells independent of their cell death control function. We used migration and invasion assays as well as three dimensional cell cultures to analyze colorectal cancer cell lines (HT29 and SW480) after siRNA mediated knockdown or overexpression of Mcl-1, Bcl-2 or Bcl-x_L_. We observed neither spontaneous cell death induction nor impaired proliferation of cells lacking Mcl-1, Bcl-2 or Bcl-x_L_. In contrast, knockdown of Mcl-1 led to increased proliferation. Strikingly, we demonstrate a profound impairment of both, migration and invasion, of colorectal cancer cells after Mcl-1, Bcl-2 or Bcl-x_L_ knockdown. This phenotype was completely revised in cells overexpressing Mcl-1, Bcl-2 or Bcl-x_L_. The most pronounced effect among the investigated proteins was observed for Bcl-2. The data presented indicate a pivotal role of Mcl-1, Bcl-2 and Bcl-x_L_ for migration and invasion of colorectal cancer cells independent of their known antiapoptotic effects. Thus, our study illustrates novel antitumoral mechanisms of Bcl-2 protein targeting.

## Introduction

Colorectal Carcinoma (CRC) is the second most common malignancy in women and the third in men worldwide with an increasing incidence. In addition, CRC is the fourth common cause of death from cancer. Even if advances in drug development and surgery led to an increased overall survival, the prognosis of patients with metastasized CRC (stage UICC IV) is still limited [1], [2]. Metastasation is a major cause of death in cancer patients and involves a multistep process of enormous complexity. Despite our growing understanding of the underlying pathways, many aspects of metastasis remain unsolved [3], [4].

The B-cell lymphoma-2 (Bcl-2) family of proteins consists of about 25 members and has been extensively studied with respect to apoptosis signaling. The delicate balance of Bcl-2 proteins governs cell’s fate at the mitochondrial surface. The proapoptotic Bcl-2 proteins (i.e. Bax and Bak) are bound by their antiapoptotic relatives (i.e. Mcl-1, Bcl-2 and Bcl-x_L_). In case of a shift of this balance towards death, the proapoptotic Bcl-2 proteins are released by their antiapoptotic counterparts. Once the proapoptotic Bcl-2 proteins are set free, mitochondria become activated and cell death occurs [5]. Furthermore, a contribution of antiapoptotic proteins to necrosis and autophagy has been shown [6], [7]. In autophagy, antiapoptotic Bcl-2 proteins act by sequestering proautophagic proteins such as Beclin1 [8], [9].

The antiapoptotic Bcl-2 proteins are widely overexpressed in human cancers including CRC. For instance, an increased expression of Bcl-x_L_ and Mcl-1 has been shown for CRCs and correlates with poor differentiation, higher tumor stage and poor prognosis of the patients [10]–[12]. In contrast, another study presents data correlating a high Bcl-2 expression with good clinical course of patients with CRC [13]. These contradictory reports point at non-redundant functions of antiapoptotic Bcl-2 proteins and elucidate the necessity for a deeper investigation of the commitment and relevance of these proteins in CRC.

There is growing evidence for a role of antiapoptotic proteins beyond cell death regulation. For instance, Mcl-1 and its splice variants have been shown to interact with the respiratory chain and the oxidative metabolism [14]. Bcl-x_L_ and Bcl-2 have been linked to signaling involved in reactive oxygen species (ROS) production [15], [16]. The effects of Bcl-2 proteins on proliferation still remain to be clarified. There is some evidence for antiproliferative effects of Bcl-2, Bcl-x_L_ and Mcl-1 in the physiological setting [17]. In this case, a survival benefit of cells less prone to apoptosis is maintained at least in part on the expense of proliferation. However, it is important to address the question, if the regulatory effects of Bcl-2 proteins on cell cycle and cell death are independent phenomena. So far, only few is known about a potential commitment of antiapoptotic Bcl-2 proteins on migration and invasiveness of cancer cells. Bcl-x_L_ has been shown to be involved in breast cancer metastasation and CRC migration, but the role of Bcl-2 and Mcl-1 to tumor spread remains unsolved [18], [19]. In our study we aimed at investigating cell death induction, proliferation, migration and invasion of CRC cells after deletion of Bcl-2, Bcl-x_L_ or Mcl-1 expression. Importantly, a knockdown of antiapoptotic Bcl-2 proteins directly inhibited migration and invasion of CRC cells independent of cell death induction or effects on proliferation. In summary, our study provides novel insights into the antitumor effects of Bcl-2 protein inhibition in colorectal cancer beyond cell death signaling and cell cycle regulation.

## Materials and Methods

### Reagents and Cell Lines

CRC cell lines HT29, SW480, CACO2 and Colo205 were purchased from ATCC. Cells were cultured in a humidified atmosphere (37°C, 5% CO_2_) in RPMI+GlutaMAX™ (Gibco, Karlsruhe, Germany) supplemented with 10% FCS (PAA Laboratories, Cölbe, Germany), 1% Pen/Strep (PAA Laboratories), 1% HEPES (Gibco) and 1% non-essential amino acids (NEAA, Gibco). All cell lines were regularly screened for contaminations and did not exceed a passage of 10. Chemotherapeutic reagents 5-Fluorouracil, Oxaliplatin and Irinotecan were purchased from Sigma-Aldrich (Hamburg, Germany).

### Viability Test

Cells were seeded onto 12 well plates and 24 h after seeding transfected or treated as indicated. Cell viability was determined using a colorimetric 3-(4, 5-Dimethylthiazol-2-yl)-2, 5-diphenyltetrazolium bromide (MTT) assay as described before [20]. Absorbance was measured at 550 nm using a microplate reader (Infinite 200 pro; Tecan, Männedorf, Switzerland).

### RNAi, Plasmids and Transfection

Small interfering RNA (siRNA) targeting Bcl-x_L_, Bcl-2, Mcl-1 mRNA or plasmid DNA was administered on day 1 after seeding of cells in 12 or 6 well plates with a confluence of 70–80% at the time of transfection. The following siRNA sequences were applied (MWG Biotech, Ebersberg, Germany): Bcl-x_L_ 5′-gcuuggauaaagaugcaaTT-3′ (sense) and 5′-uugcaucuuaucccaagcAG-3′ (antisense), Mcl-1 5′-aaguaucacagacguucucTT-3′ (sense) and 5′-gagaacgucugugauacuuTT-3′ (antisense), Bcl-2 5′-uaauaacgugccucaugaaTT-3′ (sense) and 5′-uucaugaggcacguuauuaTT-3′ (antisense). siRNA against GFP was used as control: GFP 5′-ggcuscguccaggagcgcaccTT-3′ (sense) and 5′-ggugcgcuccuggacgguagccTT-3′ (antisense). Lower case letters represent ribonucleotides and capitals deoxyribonucleotides. Cells were transfected in OptiMEM® (Invitrogen, Karlsruhe, Germany) without any supplements using RNAiMAX™ (Invitrogen) according to the manufacturer’s protocol.

Plasmid transfection was performed using Lipofectamine® LTX (Invitrogen) in OptiMEM® for SW480 cells or peqFECT DNA (Peqlab, Erlangen, Germany) in complete RPMI for HT29 cells according to the manufacturer’s protocol. The following plasmids were used: human Mcl-1 was cloned in a pEF4 vector. Human Bcl-2 and Bcl-x_L_ were cloned in a pcDNA3 vector. pcDNA3-hBCL-2 was a kind gift of W. Roth (Institute of Pathology, Heidelberg). pcDNA3-hBCL-x_L_ was kindly provided by M. Li-Weber and P.H. Krammer (German Cancer Research Center, Heidelberg, Germany). Corresponding empty vectors were used as controls. In parallel, transfection efficiency was validated using GFP tranfection by flow cytometry. Transfection efficiency was at least 70% in all experiments (data not shown) and further evaluated by Western blotting 24, 48 and 72 h after transfection.

### Detection of Proliferation and Cell Death

After treatment, supernatant was transferred to FACS tubes and cells were gently detached using Accutase™ (PAA) and pooled with the corresponding supernatant. After centrifugation, cells were resuspended in a hypotonic buffer containing 0,1% (w/v) sodium citrate, 0,1% (v/v) Triton X-100 and 50 µg/ml Propidium iodide (Sigma-Aldrich). After 1 h of incubation at 4°C total DNA content of cells was measured according to the protocol of Nicoletti *et al.* [21] using a CANTO II flow cytometer (Becton Dickinson [BD], Franklin Lakes, NJ USA). Cell cycle analysis was performed using FACS Diva 6 (BD) and FlowJo 7.6.5. (Tree Star Inc., Ashland, OR USA). Cells representing the subG1 fraction were depicted as apoptotic.

To further specify cell death induction and proliferation, cells were fixed and permeabilized using CellCycle and Proliferation kit for flow cytometry (BD) according to the manufacturer’s protocol. In brief, cells were pulsed for 1 h with 20 µM Bromodeoxyuridine (BrdU) to discriminate resting from proliferating cells. Hereafter, cells were harvested, fixed, permeabilized and then refixed followed by staining with fluorochrome coupled antibodies against cleaved PARP (Asp214, coupled to PE) as an indicator for apoptosis, BrdU (coupled to PerCP-Cy™ 5.5) for proliferation and phosphorylated-H2AX (pS139, coupled to Alexa Fluor® 647) for DNA damage, respectively. Cells were then analyzed by flow cytometry.

### Cell Lysis, SDS-Page and Western Blotting

Cells were seeded into 12 well plates, cultured for 24 h and treated as indicated. Cell lysis, SDS-Page and Western blotting were performed. The following antibodies were used for immunodetection: anti-Mcl-1 (Santa Cruz Biotechnology, Heidelberg, Germany), anti-Bcl-x_L_ (Cell Signaling, Boston, MA USA), anti-Bcl-2 (Abcam, Cambridge, UK), anti-αTubulin (Sigma), anti-cleaved PARP (ASP214, Cell Signaling).

### Cell Counting

Cells were seeded into 6 well plates and transfected with specific siRNA after 24 h. Cells were harvested, resuspended and then counted using a Neubauer chamber 24, 48 and 72 h post transfection. Trypane blue staining was used to exclude dead and fragmented cells during counting procedure.

### Migration Assays

2×10^6^ cells were seeded into 12 well plates, grown to a confluence of about 70–80% and transfected as indicated. 24 h after transfection the cell monolayer was scratched using a sterile pipette tip. Cells were then washed with medium and images were immediately captured using an inverted microscope (CKX41, Olympus Inc., Hamburg, Germany) equipped with a digital color camera (XC30, Olympus Inc.). The exact location of the image within the monolayer was marked to identify the same gap over the next 72 h. The gap closure was measured every 24 h as follows using CellSense® imaging software (Olympus Inc.): Gap distances of the scratch between one side and the other were measured at certain intervals along the edge of the generated scratch (every 200 µm). The mean of the measured distances was then calculated and compared to the mean distance of the gap at the starting time point of the experiment [22].

### Invasion Assay

BioCoat™ Matrigel™ Invasion Chambers (8 micron pore size, BD) were used to study invasion of SW480 cells. Matrigel™ invasion chambers were hydrated in FCS free RPMI in a humidified atmosphere for 2 h. Hereafter, invasion chambers were transferred in companion plate wells (BD) containing 750 µl RPMI supplemented with 10% FCS. FCS serves as a chemoattractant for invasive cells. Cells were seeded into 12 well plates and transfected as described. 24 h post transfection cells were harvested using Accutase™ (PAA), pooled and counted. 3×10^5^ cells were then resuspended in 500 µl FCS free RPMI and transferred into the upper part of the invasion chamber. After 72 h, the upper surface of the invasion chamber was scrubbed to remove non-invading cells using a cotton tipped swab. Invaded cells on the lower surface of the insert were fixed with 80% EtOH for 30 min followed by staining of the nuclei using Hoechst 33342 (Invitrogen) for 15 min. Inserts were then washed in PBS. Five pictures of every insert were taken and the number of invaded cells was counted by the naked eye.

### 3D Cell Culture

We used Alvetex® Scaffolds made of an inert, highly porous and cross-linked polystyrene scaffold, sectioned into a 200 µm thick membrane to perform suitable three dimensional cell culture experiments. Alvetex® Scaffolds were used in 12 well plate format (Reinnervate AVP002, Sedgefield, UK) under sterile conditions. Scaffolds were incubated with sterile filtered EtOH (80%) for 5 min as a pretreatment, washed twice with medium and left in medium. Cells were transfected and harvested using Accutase™ as described, counted and resuspended in complete RPMI. 1×10^6^ cells were then seeded on top of the insert disc in a total volume of 200 µl medium. 3 h after seeding, scaffolds were flooded gently from below until complete coverage was reached. Medium was changed every 48 h.

### Sectioning and Immunohistochemistry of 3D-Scaffolds

After 72 h, scaffolds were washed twice in PBS, cut into slices and immediately transferred in Cryomolds® containing OCT mounting medium (Science Services, Munich, Germany) and gradually frozen in the gas phase of liquid nitrogen. After 24 h at −80°C, the scaffolds were cryosectioned (Cryostat, Thermo) and mounted on HistoBond® slides. Sections (9 µm thickness) were fixed in 4% PFA and stained with Haematoxylin and Eosin for morphological studies, total cell count and measurement of invasion depth. In addition, immunhistochemistry was performed using NovoLink Polymer detection System (Leica Microsystems, Wetzlar, Germany) according to the manufacturer’s instructions after PFA-fixation and heat induced epitope retrieval (HIER) in citrate buffer (pH6). Antibodies against Ki67 (Abcam), and Bcl-x_L_ (Cell Signaling) were used and slides were incubated at room temperature for 30 minutes. Images were captured using an inverted microscope. Images were analyzed using CellSense® and ImageJ software.

### Statistical Analysis

Data obtained in invasion and 3D-scaffold experiments were analyzed using the Student's t-test (paired, two-sided) based on normal data distribution. For Migration Assays, the relationship between gapclosure as response, and time and treatment as explanatory variables was investigated by an analysis of variance (ANOVA). The analysis of the interaction between time and treatment was not an objective of the present study and it was not included in the variance model. SPSS 20 statistics (IBM, NY USA) software was used for all statistic analyses. A value of P<0,05 was considered to be significant.

## Results

### Mcl-1, Bcl-2 and Bcl-xL Expression in Human Colorectal Cancer Cell Lines

**Figure 1:**
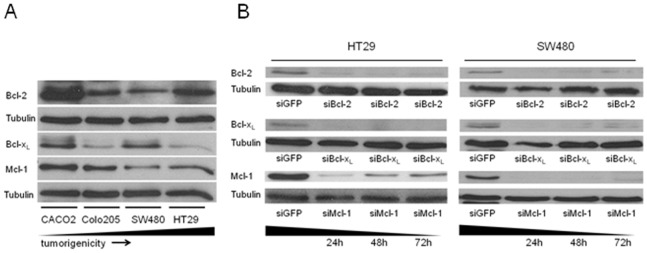
Mcl-1, Bcl-2 and Bcl-x_L_ expression and knockdown in human CRC cell lines. (**A**) Western blot analyses of the colorectal cancer cell lines CaCo2, Colo205, SW480 and HT29. Basal expression levels of Bcl-x_L_, Mcl-1 and Bcl-2. Tubulin served as loading control. Cells are arranged with increasing tumorigenicity from left to right. Here, tumorigenicity indicates the ability of a cell line to metastasize in mice. The lowest tumorigenicity stands for local tumor growth lacking invasiveness; the highest tumorigenicity indicates the formation of distant organ metastasis (liver and/or lung). (**B**) Western blot analyses of HT29 (left) and SW480 (right) cells after siRNA mediated knockdown of Mcl-1, Bcl-2 and Bcl-x_L_ 24, 48 and 72 h post transfection. Tubulin served as loading control. The Western blots presented are representative of three independent experiments.

In order to investigate the expression of the antiapoptotic Bcl-2 proteins Mcl-1, Bcl-2 and Bcl-x_L_ in human CRC cell lines, we measured protein levels in four colorectal cancer cell lines. CaCo2, Colo205, HT29 and SW480 cells are widely used and well characterized [23], [24]. CaCo2 cells are known to be poorly tumorigenic in mice. SW480 and Colo205 cells are locally invasive in orthotopic tumor models. HT29 cells metastasize in liver and lung when injected in the subcutis [24]. Mcl-1, Bcl-2 and Bcl-x_L_ expression was detectable in CRC cell lines (Fig. 1A). CaCo2 cells showed considerable amounts of Mcl-1, Bcl-2 and Bcl-x_L_, with the highest expression level for Bcl-2. Colo205 cells had a decreased amount of Bcl-x_L_. SW480 cells had an approximately equal amount of all Bcl-2 proteins. HT29 cells showed a dominant expression of Bcl-2 and a low level of Bcl-x_L_. Taken together, these findings demonstrate that the expression pattern of antiapoptotic proteins varied in the CRC cell lines used in our study. There was no correlation between tumorigenicity of a cell line and a certain expression pattern (Fig. 1A). We decided to focus on SW480 cells and HT29 for further experiments with Bcl-2 knockdown, because both cell lines share the ability to invade and form metastasis in mouse models.

### Knockdown of Antiapoptotic Bcl-2 Proteins does not Induce Spontaneous Apoptosis but Sensitizes CRC Cells to Chemotherapy

**Figure 2:**
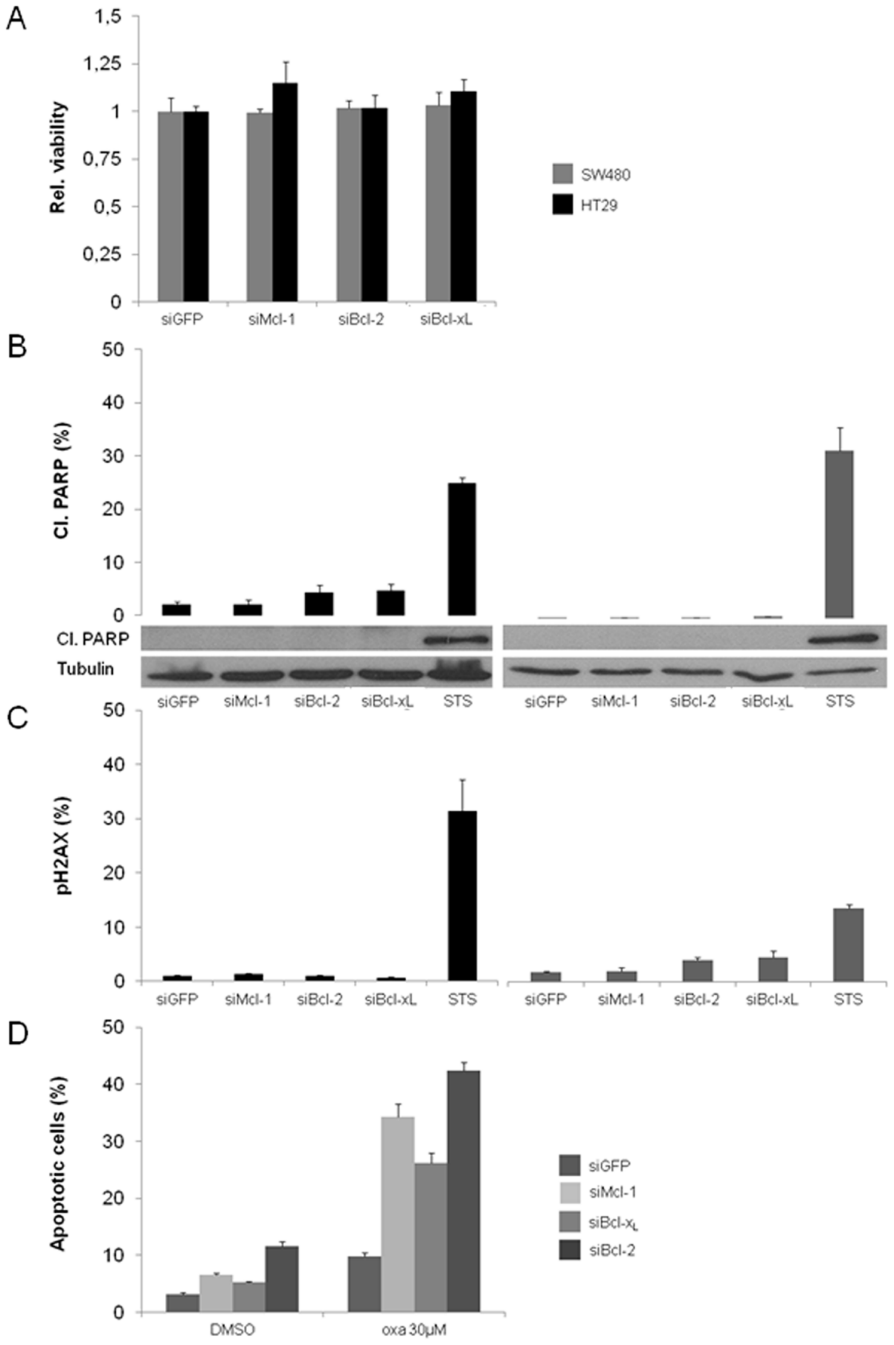
Viability, cell death and chemosensitization after siRNA mediated knockdown of Mcl-1, Bcl-2 and Bcl-xL. (**A**) MTT-Assay of SW480 and HT29 cells after knockdown of Mcl-1, Bcl-2 and Bcl-x_L_. (**B**) Flow cytometric analyses and corresponding Western blots for cleavage of PARP 48 h after knockdown of Mcl-1, Bcl-2 and Bcl-x_L_ in HT29 (left) and SW480 cells (right). (**C**) Flow cytometric analysis of HT29 (left) and SW480 cells (right) for pH2AX 48 h after knockdown of Mcl-1, Bcl-2 and Bcl-x_L_. 24 h Staurosporine treatment (1 µM) served as a positive control for cell death induction. (**D**) Flow cytometric analysis for DNA-fragmentation of HT29 cells after knockdown of Mcl-1, Bcl-2 and Bcl-x_L_ followed by 48 h treatment with 30 µM oxaliplatin and 0,2% DMSO as a vehicle. Flow cytometry analysis was performed in triplicates. Bars represent mean ± SD. Assays are representative of at least three independent experiments. (oxa = oxaliplatin).

We first determined the effect of specific siRNA targeting Bcl-2, Bcl-x_L_ or Mcl-1 over a time period of 72 h in HT29 and SW480 cells. Here, the downregulation of antiapoptotic proteins was efficient for at least 72 h as observed by Western blot analysis (Fig. 1 B). In order to analyze the impact of a downregulation of Bcl-2, Bcl-x_L_ or Mcl-1 on cell viability we performed MTT assays 48 h post transfection. The results indicate no effects of decreased antiapoptotic Bcl-2 protein levels on cell viability in HT29 and SW480 cells (Fig. 2 A).

Second, we investigated whether spontaneous cell death induction occurred in transfected HT29 and SW480 cells. Therefore, we analyzed cells for cleaved poly (ADP-ribose) polymerase (PARP) 48 h after transfection by flow cytometry as well as by Western blotting. Since PARP is a target of activated Caspase 3 its cleavage indicates an efficient execution of apoptosis [25]. We did not detect an increase in cleaved (cl.) PARP levels, subsequent to Mcl-1 knockdown (Fig. 2 B). For Bcl-2 and Bcl-x_L_ knockdown, we detected a very slight increase in cl. PARP levels by FACS, but could not detect a reasonable amount of cleaved protein in Western blots (Fig. 2 B). In addition, we checked for DNA damage as a hallmark of cell death. Therefore, we stained cells 48 h after transfection with fluorochrome coupled antibody targeting phosphorylated histone H2AX [26]. Phosphorylation of H2AX occurs in response to DNA double strand breaks. There was no considerable amount of phosphorylated H2AX detectable in transfected cells and controls (Fig. 2 C). Staurosporine (STS) treatment of HT29 and SW480 cells served as a positive control for cell death induction (Fig. 2 B and C).

Third, we aimed to investigate the potential of antiapoptotic Bcl-2 protein knockdown to sensitize HT29 cells to clinically relevant and commonly used chemotherapeutics. For 5-FU and irinotecan there were no significant sensitizing effects of siRNA mediated knockdown of antiapoptotic proteins, as assessed by FACS analysis of DNA fragmentation (data not shown). In striking contrast, we observed a profound and highly significant sensitization to oxaliplatin after knockdown of the antiapoptotic proteins Bcl-2, Bcl-x_L_ and Mcl-1 (Fig. 2D).

Taken together, these results indicate that a knockdown of Bcl-2, Bcl-x_L_ or Mcl-1 did not lead to spontaneous cell death and was apparently well compensated in CRC cells. Among the chemotherapeutics applied in CRC treatment, only the effect of oxaliplatin was markedly augmented by knockdown of antiapoptotic proteins.

### Knockdown of Antiapoptotic Bcl-2 Proteins Exerts no Antiproliferative Effects on CRC Cells

**Figure 3:**
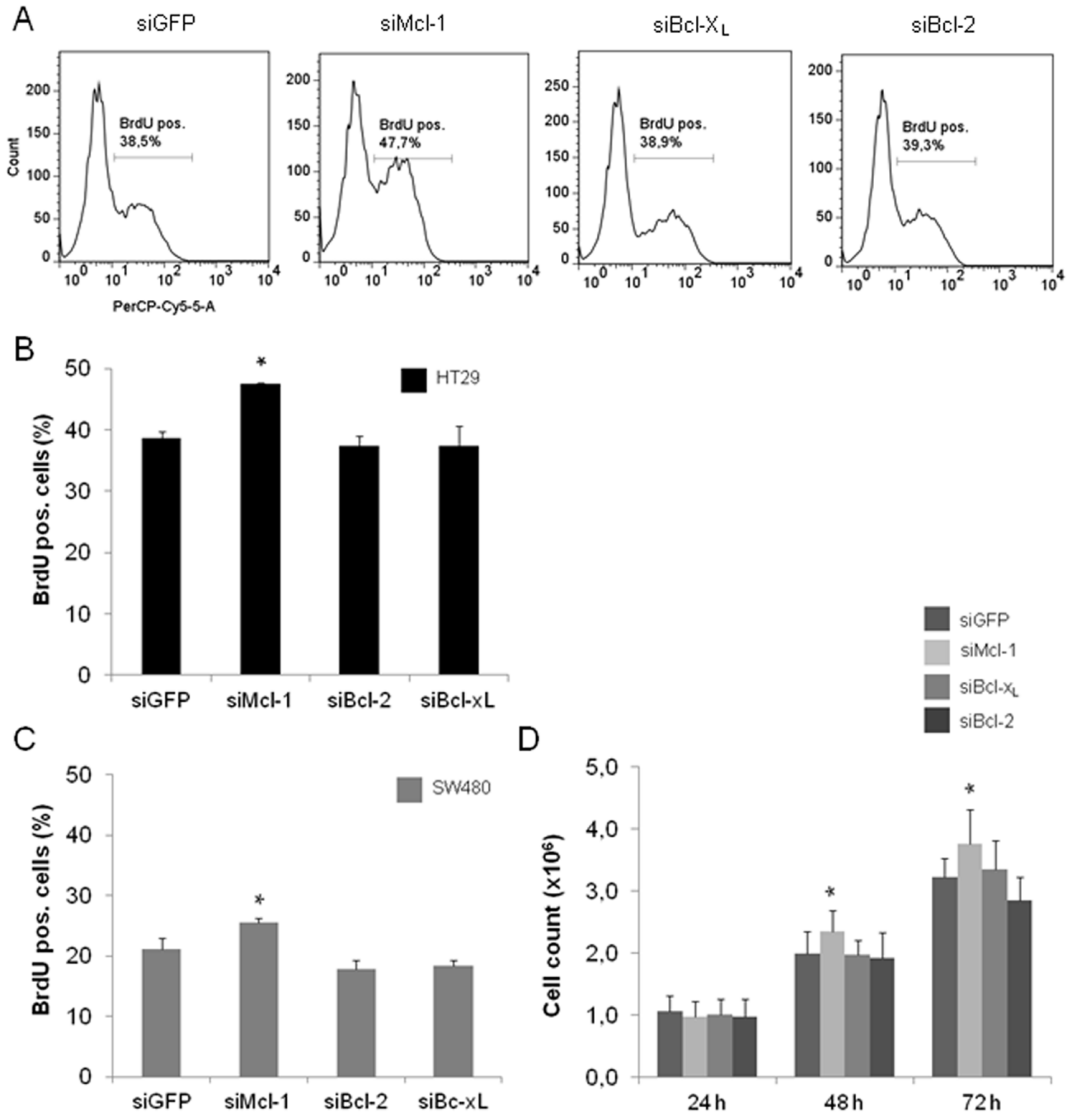
Proliferation of SW480 and HT29 cells after siRNA mediated knockdown of Mcl-1, Bcl-2 and Bcl-xL. SW480 and HT29 cells were transfected with siRNA against Mcl-1, Bcl-2 and Bcl-x_L_. 24 h post transfection, cells were pulsed with 20 µM BrdU and prepared for flow cytometry. (**A**) Representative original flow cytometry data with HT29 cells for BrdU positivity after staining with anti-BrdU antibody coupled to PerCP-CY5.5 fluorophore. (**B**) Flow cytometric analyses for BrdU incorporation in HT29 and (**C**) SW480 cells. (**D**) Total cell count of SW480 cells after knockdown of Mcl-1, Bcl-2 and Bcl-x_L_. Cells were seeded on 6 well plates, harvested and counted 24, 48 and 72 h post transfection. Values are expressed as means ± SD. Assays were run in triplicates (flow cytometry) and sextuplicates (cell counting). Assays are representative of at least three independent experiments. *p<0,05.

After exclusion of spontaneous cell death induction after Bcl-2, Bcl-x_L_ or Mcl-1 knockdown we next investigated proliferation of CRC cells. A high proportion of untreated HT29 cells (38,6%) was proliferating as assessed by BrdU incorporation (Fig. 3 A and B). The ratio of proliferating cells was not significantly different after Bcl-2 and Bcl-x_L_ knockdown (siBcl-2∶37,3%; siBcl-x_L_: 37,4%). In contrast, knockdown of Mcl-1 led to a significant increase in BrdU incorporation in comparison to controls (47,5 vs. 38,6%, p<0,05, Fig. 3 A and B). For SW480 cells, we observed a lower basal level of proliferation (21,1% BrdU positive cells). The results for cells transfected with siRNA against Mcl-1, Bcl-2 and Bcl-x_L_ were in line with those obtained for HT29 cells. Again, only Mcl-1 knockdown led to a significant increase of BrdU positivity, whereas Bcl-2 and Bcl-x_L_ knockdown showed no significant effects (Mcl-1∶25,6% vs. 21,1%, p<0,05, Fig. 3 C). To further validate our results on proliferation after siRNA transfection, we followed total cell numbers of SW480 cells over time. The results were in line with the BrdU experiments: Only deletion of Mcl-1 led to an increase of total cell counts, whereas no significant changes were observed for Bcl-2 and Bcl-x_L_ deletion (Fig. 3 D). Taken together, our data indicate that there is no significant effect of a knockdown of Bcl-2 and Bcl-x_L_ on proliferation. By contrast, a lack of Mcl-1 leads to increased proliferation pointing at specific functions of Mcl-1 in this context.

### Knockdown of Antiapoptotic Bcl-2 Proteins Massively Impairs Migration of CRC Cells

**Figure 4:**
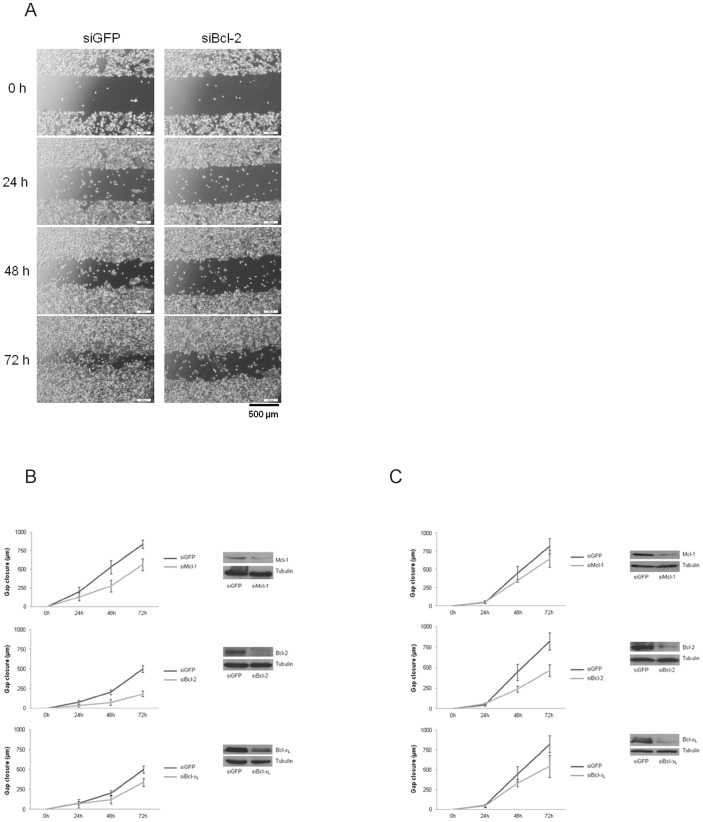
Migration of SW480 and HT29 cells after siRNA mediated knockdown of Mcl-1, Bcl-2 and Bcl-xL. SW480 and HT29 cells were transfected with siRNA against Mcl-1, Bcl-2 and Bcl-x_L_ and grown as a monolayer. Gap distance was measured every 200 µm along the edge of the gap and gap closure calculated 24, 48 and 72 h post transfection. (**A**) Representative pictures captured after knockdown of Bcl-2 in HT29 cells (scale bar indicate magnification for all panels). (**B**) Gap closure kinetics of HT29 cells after knockdown of Mcl-1, Bcl-2 and Bcl-x_L_ (left) and corresponding Western blots (right). (**C**) Gap closure kinetics of SW480 cells after knockdown of Mcl-1, Bcl-2 and Bcl-x_L_ (left) and corresponding Western blots (right). Assays are representative of at least three independent experiments. Values are expressed as mean ± SD. (p-values for HT29: siMcl-1:<0,001; siBcl-2:<0,001; siBcl-x_L_: = 0,002. P-values for SW480: siMcl-1: = 0,0011; siBcl-2: = 0,0005; siBcl-x_L_: = 0,004).

Next, we aimed to investigate effects of Mcl-1, Bcl-2 and Bcl-x_L_ deletion on the migration of CRC cells. In order to visualize cell migration, we performed scratch assays on monolayers of transfected HT29 and SW480 cells. Gap distance was measured every 24 h after knockdown of Mcl-1, Bcl-2 and Bcl-x_L_ followed by calculation of gap closure. Gap closure was significantly slowed down in both HT29 and SW480 cells after knockdown of Mcl-1, Bcl-2 and Bcl-x_L_ (p-values for HT29: siMcl-1:<0,001; siBcl-2:<0,001; siBcl-x_L_: = 0,002. P-values for SW480: siMcl-1: = 0,0011; siBcl-2: = 0,0005; siBcl-x_L_: = 0,004. ) (Fig. 4 B and C, left). In HT29 and SW480 cells, the most striking effect was observed after knockdown of Bcl-2. Here, the migration distance was decreased to 56% in SW480 and 36% in HT29 after 72 h compared to controls (Fig. 4 A; Fig. 4 B and C, left). Taken together, we demonstrate negative effects of a Mcl-1, Bcl-x_L_ and Bcl-2 knockdown on CRC migration independent of cell death induction and antiproliferative effects.

### Overexpression of Antiapoptotic Bcl-2 Proteins Accelerates Migration of CRC Cells

**Figure 5:**
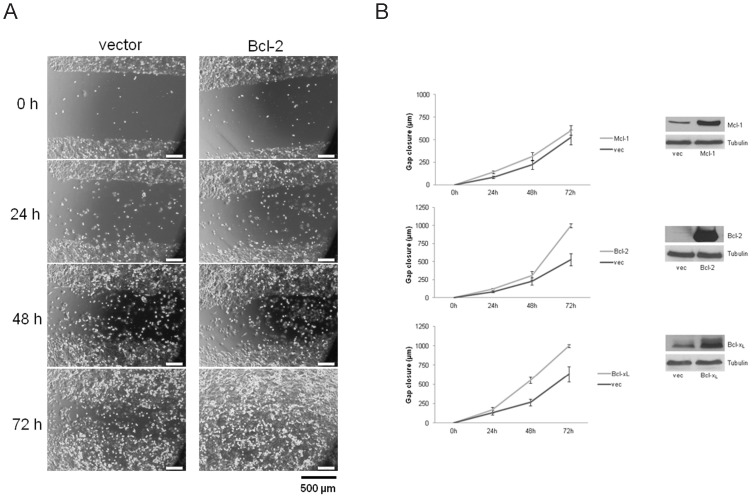
Migration of Mcl-1, Bcl-2 or Bcl-xL overexpressing SW480 cells. SW480 cells were transfected with plasmids expressing human Mcl-1, Bcl-2 or Bcl-x_L_ and grown as a monolayer. Gaps were generated and gap closure measured as described. (**A**) Representative pictures for SW480 cells overexpressing Bcl-2 (scale bar indicates magnification for all panels). (**B**) Gap closure kinetics of SW480 cells overexpressing Mcl-1, Bcl-2 and Bcl-x_L_ (left) and corresponding Western blots (right). Assays are representative of at least three independent experiments. Values are expressed as mean ± SD. p-values: Mcl-1: = 0,0006; Bcl-2: = 0,0002; Bcl-x_L_:<0,0001.

In addition, we tested whether increased expression of Mcl-1, Bcl-2 and Bcl-x_L_ in HT29 and SW480 cells influences migration and potentially reverts the phenotype of the knockdown experiments. Again, we performed scratch assays and used cells transfected with corresponding expression plasmids of Bcl-2 proteins. Strikingly, we observed a significantly accelerated migration of SW480 cells overexpressing Bcl-x_L_ and Bcl-2 (p<0,001, Fig. 5 B, left). Cells overexpressing Bcl-2 almost doubled migrated distance after 72 h (999 µm vs. 526 µm in controls, Fig. 5 A). For Mcl-1 overexpressing SW480 cells we observed a lower, but significant increase in migration (p<0,01, Fig. 5 B, left). These observations were also made for HT29 cells with overexpression of Mcl-1, Bcl-2 or Bcl-x_L_ (data not shown). Furthermore, there was no increased proliferation detectable in cells overexpressing Mcl-1, Bcl-2 or Bcl-x_L_ (data not shown). In summary, we show that antiapoptotic Bcl-2 proteins are capable of triggering migration of CRC cells.

### Knockdown of Antiapoptotic Bcl-2 Proteins Regulate Invasiveness of CRC Cells

**Figure 6:**
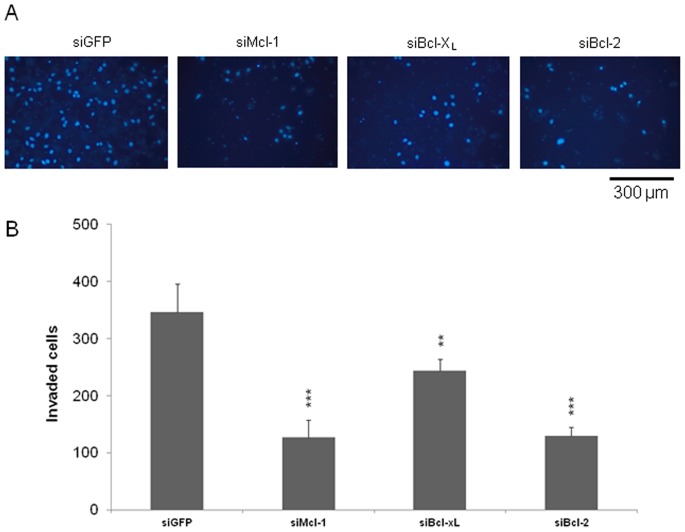
Invasion of SW480 cells after siRNA mediated knockdown of Mcl-1, Bcl-2 and Bcl-xL. SW480 cells were seeded on 6 well plates and transfected as described. 24×10^5^ cells were seeded into the upper chamber of a transwell. 48 h after seeding, nuclei on the lower surface were visualized by Hoechst staining. (**A**) Representative pictures of lower insert surface after Hoechst staining (scale bar indicates magnification for all panels). (**B**) Five fields of view per insert were counted. n = 5 per group. Values are expressed as mean ± SD. Assays are representative of at least three independent experiments. **p<0,01. ***p<0,001.

To further investigate regulatory effects of Mcl-1, Bcl-x_L_ and Bcl-2 on processes relevant for CRC metastasis, we also assessed invasion of CRC cells after manipulation of Bcl-2 proteins. Cells with deleted Bcl-x_L_ showed significantly impaired invasive properties in boyden chamber invasion assays compared to mock transfected cells (70% compared to controls, p<0,05; Fig. 6 A and B). Most strikingly, a knockdown of Bcl-2 almost completely abrogated the capacity of SW480 cells to invade (38% compared to controls, p<0,001). In addition, Mcl-1 knockdown also caused a profound decrease of invasion (37% compared to controls, p<0,001; Fig. 6 A and B). These observations underline the role of antiapoptotic Bcl-2 proteins for migration and invasion of CRC cells and identify a prominent role of Bcl-2 in the context of invasiveness.

### Knockdown of Mcl-1, Bcl-2 and Bcl-xL Decreases Motility of CRC Cells in 3D- Culture Models

**Figure 7:**
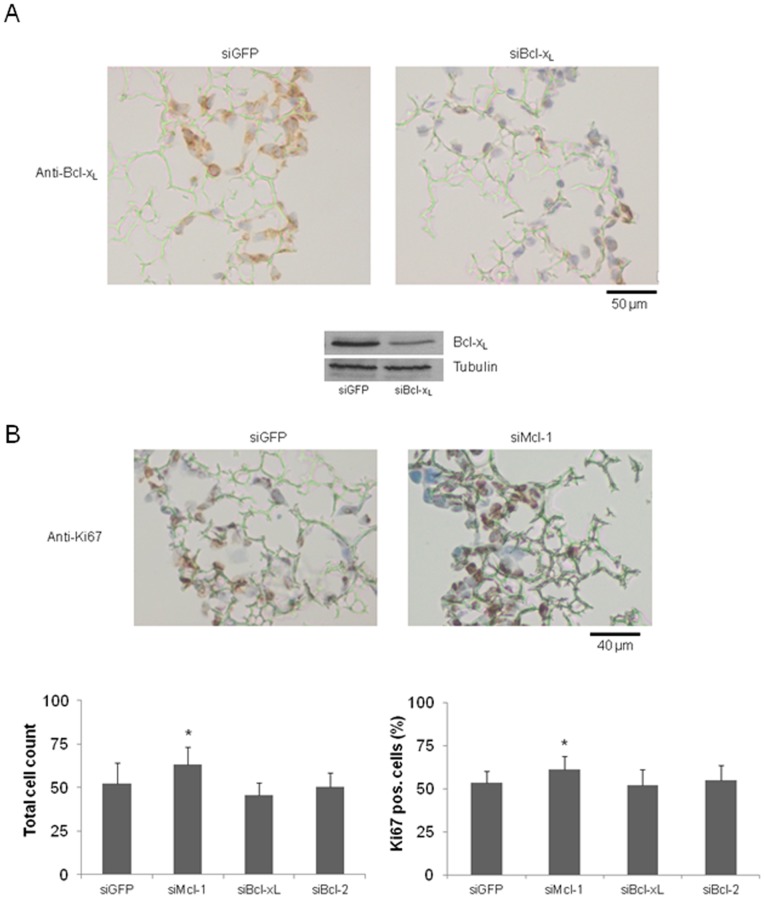
Proliferation of HT29 cells in three dimensional scaffolds after siRNA mediated knockdown of Mcl-1, Bcl-2 and Bcl-xL. HT29 cells were transfected with specific siRNA against Mcl-1, Bcl-2 or Bcl-x_L_. 24 h post transfection, cells were harvested and 1×10^6^ cells were seeded on each scaffold. After 72 h, scaffolds were further processed for Western blotting or immunohistochemistry. (**A**) Representative pictures of scaffolds after siRNA mediated knockdown of Bcl-x_L_ and corresponding Western blot. (**B**) Total cells in scaffolds were counted (n = 5 per group, lower left graph). Scaffolds were stained for Ki67 (representative pictures for knockdown of Mcl-1 and lower right graph, scale bar indicates magnification for all panels, n = 5 per group). Values are expressed as mean ± SD. Assays are representative of at least three independent experiments. *p<0,05.

**Figure 8:**
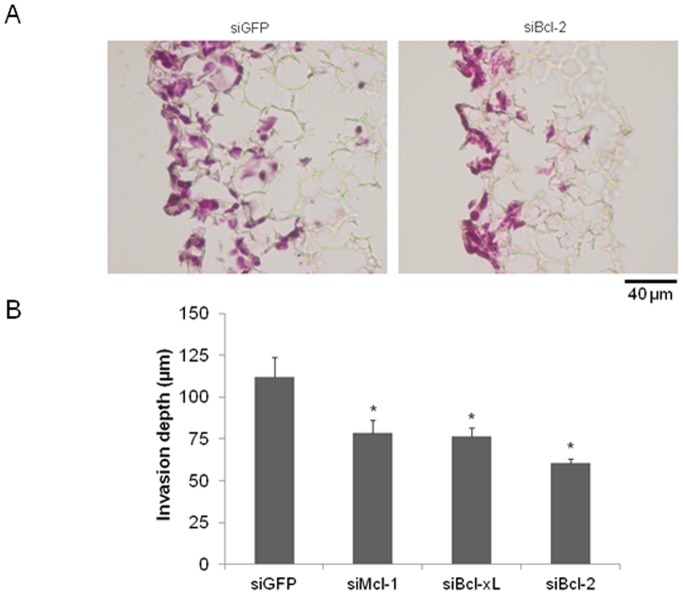
Migration of HT29 cells in three dimensional scaffolds after siRNA mediated knockdown of Mcl-1, Bcl-2 and Bcl-xL. HT29 cells were transfected with specific siRNA against Mcl-1, Bcl-2 or Bcl-x_L_. 24 h post transfection, cells were harvested and 1×10^6^ cells were seeded on each scaffold. (**A**) Haematoxylin and Eosin staining of transfected cells in scaffolds (representative pictures for knockdown of Bcl-2 in scaffolds, scale bar indicates magnification for both pictures). (**B**) Invasion depth was measured every 20 µm (n = 5 per group). Values are expressed as mean ± SD. Assays are representative of at least three independent experiments. *p<0,05.

Finally, we aimed to validate our data on migration and invasion in a 3D cell culture model based on a polystyrene scaffold. These scaffolds facilitate cell-cell as well as cell-matrix interactions thereby reflecting a more physiological environment compared to conventional 2D culture models. Additionally in 3D cell culture, HT29 cells showed high transfection efficiency measured via Western blotting and immunohistochemical staining. For example, the application of siRNA against Bcl-x_L_ mRNA significantly reduced the ratio of Bcl-x_L_ positive cells (20% compared to control transfected cells; Fig. 7A). Since we expected a general increase in proliferation in scaffolds due to the three dimensional environment, we first aimed to analyze proliferation after sectioning of the scaffolds. We found no significant changes in total cell counts after knockdown of Bcl-x_L_ and Bcl-2. In contrast, Mcl-1 knockdown led to a significant increase in total cell count (an average of 63 cells per visual field vs. 52 cells in controls, p<0,05; Fig. 7 B, lower left graph). To validate these findings, we performed immunohistochemical stainings of Ki67 as an additional approach to assess proliferation. We observed no significant changes in Ki67 positivity after knockdown of either Bcl-2 or Bcl-x_L_. Knockdown of Mcl-1, however, led to a significant increase in proliferation (53 vs. 61% of cells positive for Ki67 in controls; Fig. 7 B, lower right graph). Representative pictures of Ki67 stained scaffolds are shown in Figure 7 B (upper part) for siMcl-1 transfected HT29 cells. We further focused on the invasive capacity of CRC cells after knockdown of antiapoptotic Bcl-2 proteins in 3D scaffolds. With regard to morphology, cells maintained their normal shape and size after knockdown of Mcl-1, Bcl-2 and Bcl-x_L_. We observed a significant decrease in the total area occupied by the cells after knockdown of all investigated proteins. The smaller size of the invaded area and a decrease in invaded distances indicated impaired migration (Fig. 8, graph and representative picture of scaffolds for siBcl-2). Again, the most striking effect was observed after knockdown of Bcl-2 (54% invaded distance compared to controls). These results are especially intriguing, since we excluded proliferation effects in the same analyzed scaffold.

## Discussion

The Bcl-2 family of proteins has been extensively studied through the past two decades leading to identification of the antiapoptotic family members as promising anticancer drug targets. Importantly, deeper mechanistic and structural insights led to the development of small molecule inhibitors targeting antiapoptotic Bcl-2 proteins, so called BH-3 mimetics [27], [28]. Despite the fact that those inhibitors have already entered clinical trials, very little is known concerning functions of Bcl-2 proteins beyond their cell death regulating properties [29], [30].

The major aim of this study was to investigate the relevance of Mcl-1, Bcl-x_L_ and Bcl-2 for migration and invasion of CRC cells *in vitro*. We first knocked out the expression of these proteins in CRC cell lines by RNA interference. Notably, assessment of spontaneous cell death or decreased viability, caused by the deletion of Bcl-2 proteins, is mandatory prior to study migration and invasion. This is because a cell affected by cell death stimuli or challenged with impaired metabolism, has to be considered as a priori less prone to migrate and invade. With this in mind, we first decided to study viability and cell death in CRC cells after knockdown of antiapoptotic Bcl-2 proteins. Our data indicate no decrease in cell viability. By contrast, cells lacking Mcl-1 showed a slightly higher viability. Second, we aimed at investigating spontaneous cell death induction in cells lacking Mcl-1, Bcl-x_L_ or Bcl-2. The key function of the Bcl-2 family in apoptosis regulation is well known [5]. In addition, there is emerging evidence for a direct function of Bcl-2 proteins on necrotic death [7]. In line with our data on viability, no reasonable amount of cell death was measured after knockdown of Mcl-1, Bcl-x_L_ or Bcl-2 in CRC cells.

Next, we focused on proliferation of CRC cells after knockdown of Mcl-1, Bcl-x_L_ or Bcl-2. Cancer cells frequently harbor defects in cell cycle regulation, which contribute to uncontrolled and sustained proliferation as a key feature of malignancy [31]. The function of antiapoptotic Bcl-2 proteins on cell cycle and proliferation remains controversial [32]. In the physiological situation, antiapoptotic Bcl-2 proteins seem to exert antiproliferative functions by delaying cell cycle progression and causing cell cycle arrest [17], [32]. Bcl-2 and Bcl-x_L_ delay G0–G1 transition and effectively arrest cells in the G-phase. The role of Mcl-1 for cell cycle regulation is apparently different and is related to the S-Phase [33], [34]. However, an important question to address is, whether a knockdown of a primary antiapoptotic protein directly affects proliferation of CRC cells. Our data presented in this study clearly show that Bcl-2 and Bcl-x_L_ have no significant effects on proliferation and cell cycle progression in CRC cells. Since Mcl-1 knockdown led to an increase in proliferation as indicated by higher total cell counts, enhanced BrdU incorporation and increased levels of Ki67, we conclude that Mcl-1 itself exerts antiproliferative functions. This finding is in line with other studies raising the hypothesis that a protection towards death acquired through a high level of Mcl-1 is at least partly maintained on the expense of proliferation [33].

It has been shown that inhibition of antiapoptotic Bcl-2 proteins in various cancer entities, including CRC, causes a sensitization to anticancer drugs [10], [20]. Here, we demonstrate that a knockdown of Mcl-1, Bcl-x_L_ or Bcl-2, profoundly sensitized CRC cells to oxaliplatin-induced cell death. In contrast, 5-FU and irinotecan did not synergistically act with a knockdown of antiapoptotic Bcl-2 proteins. The mechanisms of the opposing synergistic effects remain elusive. In contrast to 5-FU and irinotecan, oxaliplatin causes DNA double strand breaks. Since there is evidence for a direct effect of Bcl-2 and other family members on DNA repair [35], we hypothesize that impaired DNA repair after knockdown of Bcl-2 proteins caused the synergistic effects of oxaliplatin and Bcl-2 protein inhibition. These synergistic effects need further attention and may help to develop novel treatment strategies in oncology.

Our results indicate that a single knockdown of either Mcl-1 and Bcl-x_L_ or Bcl-2 is quite well compensated in CRC cells with regard to cell death and proliferation. Our data regarding viability, spontaneous cell death and proliferation after knockdown of antiapoptotic Bcl-2 proteins build up the basis to further study migration and invasion: Antiproliferative effects and spontaneous cell death induction could be excluded as a potential cause for changes in migration and invasion capacities. Therefore, we proceeded with carrying out in depth experiments on the migrative and invasive phenotype of CRC cells. Migration and invasion are fundamental processes for the capacity of tumor cells to metastasize [36]. Even if the formation of metastasis is a highly complex and multistep process, the contribution of an early acquired apoptosis resistance to metastasis is well documented [37]. The propensity of a cancer cell to metastasize has been linked to mechanisms of apoptosis resistance [38]. However, a direct contribution of antiapoptotic Bcl-2 proteins to migration and invasion of colorectal cancer has not been investigated so far.

We observed a decreased migration after knockdown of Mcl-1, Bcl-x_L_ and Bcl-2. For both investigated cell lines (HT29 and SW480), Bcl-2 knockdown caused the most striking inhibition of gap closure in wound healing scratch assays. This phenotype was completely reversed in cells overexpressing Mcl-1, Bcl-x_L_ or Bcl-2. Impressively, overexpression of every single protein led to an enhanced migration of CRC cells. In line with these findings, other groups demonstrated that high levels of Bcl-2 and Bcl-x_L_ attributed to highly metastatic behavior of several cancer cells including CRC [18], [39]. To further validate the observed migration phenotype, we used three dimensional scaffolds, which foster cell-cell contacts and allow cells to migrate and proliferate more freely [40], [41]. These 3D cultures better reflect the pathophysiological situation of cancer cell growth compared with 2D culture systems [42]. We found a severely impaired migration of cells in 3D after knockdown of Mcl-1, Bcl-x_L_ and Bcl-2. Again, Bcl-2 knockdown caused the most robust and intense inhibition of migration. It is noteworthy that cells were morphologically unaltered after knockdown of Mcl-1, Bcl-x_L_ and Bcl-2.

A cancer cell leaving the primary tumor penetrates the extracellular matrix (ECM) as a first obstacle on its way to form distant metastases [43]. To mimic the *in vivo*-situation, we included trans-well invasion assays in which cells penetrate a Matrigel™ layer mimicking the ECM. Our data indicate that Bcl-2 and Mcl-1 knockdown caused an almost complete abrogation of invasiveness of CRC cells. In addition, Bcl-x_L_ knockdown also profoundly inhibited invasion to an impressive extent. These findings are in line with data showing that ectopic expression of antiapoptotic Bcl-2 proteins leads to enhanced Matrigel™ invasion of glioma cells [44]. Moreover, there is experimental evidence for a correlation between a decreased metastatic potential with lowered Bcl-2 levels in prostate cancer [45], [46]. Another study on prostate cancer revealed that an antitumor growth effect of Nuclear Factor kappa B (NF-κB) suppression in mice is crucially dependent on downregulation of Bcl-2 and Bcl-x_L_ [47]. In addition, Bcl-2 overexpression increased the metastatic potential in human breast cancer cell lines [48]. Bcl-x_L_ overexpression correlates with nodal involvement and a more aggressive tumor in breast cancer patients [49]. Both, Bcl-2 and Bcl-x_L_, exert effects on distant organ metastasis rather than on the primary breast tumor [18], [49], [50]. Adenoviral-mediated depletion of Bcl-x_L_ resulted in reduced migration and invasion, but also induced apoptosis in CRC cells [19]. So far, there is no study addressing the role of Bcl-2 on the metastasis in colorectal cancer. Furthermore, regarding the effects of Mcl-1 on invasion, there is no supportive experimental data available on any cancer entity so far. Our data indicate that the migration and invasion inhibitory phenotype of a knockdown of Mcl-1, Bcl-x_L_ and Bcl-2 in CRC cells is a shared feature of all investigated Bcl-2 proteins. Remarkably, the inhibition of migration and invasion after knockdown of Bcl-2 proteins is independent of a cell cycle regulation and a cell death induction.

## Conclusions

Based on this study we conclude that Mcl-1, Bcl-2 and Bcl-x_L_ contribute to migration and invasion of colorectal cancer cells independent of their antiapoptotic effects. Our study highlights the need to further develop treatment strategies, which include targeting of anti-apoptotic Bcl-2 proteins in CRC.
